# Minimally invasive surgeries for spontaneous hypertensive intracerebral hemorrhage (MISICH): a multicenter randomized controlled trial

**DOI:** 10.1186/s12916-024-03468-y

**Published:** 2024-06-13

**Authors:** Xinghua Xu, Huaping Zhang, Jiashu Zhang, Ming Luo, Qun Wang, Yining Zhao, Zhichao Gan, Bainan Xu, Xiaolei Chen, the MISICH study team

**Affiliations:** 1 Department of Neurosurgery, The First Medical Center, Chinese PLA General Hospital, 28 Fuxing Road, Beijing, 100853 China; 2 Department of Neurosurgery, Jingzhou Central Hospital, Hubei, China; 3 Department of Neurosurgery, Wuhan No.1 Hospital, Hubei, China; 4 Department of Neurosurgery, Erlangen-Nuernberg University Hospital, Erlangen, Germany

**Keywords:** Intracerebral hemorrhage, Minimally invasive surgery, Endoscopic surgery, Stereotactic aspiration, Small-bone flap craniotomy, Randomized controlled trial

## Abstract

**Background:**

Intracerebral hemorrhage (ICH) is a common stroke type with high morbidity and mortality. There are mainly three surgical methods for treating ICH. Unfortunately, thus far, no specific surgical method has been proven to be the most effective. We carried out this study to investigate whether minimally invasive surgeries with endoscopic surgery or stereotactic aspiration (frameless navigated aspiration) will improve functional outcomes in patients with supratentorial ICH compared with small-bone flap craniotomy.

**Methods:**

In this parallel-group multicenter randomized controlled trial conducted at 16 centers, patients with supratentorial hypertensive ICH were randomized to receive endoscopic surgery, stereotactic aspiration, or craniotomy at a 1:1:1 ratio from July 2016 to June 2022. The follow-up duration was 6 months. Patients were randomized to receive endoscopic evacuation, stereotactic aspiration, or small-bone flap craniotomy. The primary outcome was favorable functional outcome, defined as the proportion of patients who achieved a modified Rankin scale (mRS) score of 0–2 at the 6-month follow-up.

**Results:**

A total of 733 patients were randomly allocated to three groups: 243 to the endoscopy group, 247 to the aspiration group, and 243 to the craniotomy group. Finally, 721 patients (239 in the endoscopy group, 246 in the aspiration group, and 236 in the craniotomy group) received treatment and were included in the intention-to-treat analysis. Primary efficacy analysis revealed that 73 of 219 (33.3%) in the endoscopy group, 72 of 220 (32.7%) in the aspiration group, and 47 of 212 (22.2%) in the craniotomy group achieved favorable functional outcome at the 6-month follow-up (*P* = .017). We got similar results in subgroup analysis of deep hemorrhages, while in lobar hemorrhages the prognostic outcome was similar among three groups. Old age, deep hematoma location, large hematoma volume, low preoperative GCS score, craniotomy, and intracranial infection were associated with greater odds of unfavorable outcomes. The mean hospitalization expenses were ¥92,420 in the endoscopy group, ¥77,351 in the aspiration group, and ¥100,947 in the craniotomy group (*P* = .000).

**Conclusions:**

Compared with small bone flap craniotomy, endoscopic surgery and stereotactic aspiration improved the long-term outcome of hypertensive ICH, especially deep hemorrhages.

**Trial Registration:**

ClinicalTrials.gov Identifier: NCT02811614.

**Supplementary Information:**

The online version contains supplementary material available at 10.1186/s12916-024-03468-y.

## Background

Stroke is a leading cause of mortality and disability worldwide, and the economic costs of treatment and poststroke care are substantial [1, 2]. Although the prevalence of intracerebral hemorrhage (ICH) is only approximately one-quarter that of ischemic stroke, the cause-specific death and years of life lost due to ICH exceed those of ischemic stroke [3–5]. Death from ICH is estimated to occur in 30% to 40% of cases, and most patients who survive ICH have disabilities and are at risk for recurrent stroke [6]. The incidence of ICH increases sharply with age and is therefore expected to remain substantial as the population ages, even with improvements in blood pressure control [7].

Theoretically, surgery has the advantages of reducing hematoma volume and relieving perihematomal edema compared with conservative treatment [8, 9]. However, findings from randomized controlled trials (RCTs) have shown that for most patients with supratentorial ICH, the effectiveness of craniotomy for hemorrhage evacuation to improve functional outcome or mortality is uncertain [10, 11]. An important factor that affects the effectiveness of craniotomy is the damage to normal brain tissue caused by surgery. Compared with conventional craniotomy, minimally invasive surgery for ICH can minimize the disruption of healthy brain tissue. Two main types of minimally invasive surgery have been attempted for hematoma removal: endoscopic surgery and stereotactic aspiration [12, 13]. To date, except for some retrospective studies or meta-analyses [14–16], no large-scale RCT comparing these three surgical interventions have been reported. Therefore, we designed the minimally invasive surgeries for spontaneous hypertensive intracerebral hemorrhage (MISICH) trial to investigate whether endoscopic surgery and/or stereotactic aspiration (frameless navigated aspiration) would improve functional outcomes in patients with supratentorial ICH compared with small-bone flap craniotomy.

## Methods

### Study design

MISICH is a multicenter, randomized controlled, open-label, sequentially designed nonprofit study (ClinicalTrials.gov ID: NCT02811614) involving 16 neurosurgical centers across China. The study followed the Consolidated Standards of Reporting Trials (CONSORT) reporting guidelines and was in accordance with the Declaration of Helsinki. The study protocol was approved by the ethics committees of the Chinese PLA General Hospital (S2016-074–01). Details about the trial design, conduct, and oversight can be found in the trial protocol in Additional file 1 and published elsewhere [17].

### Participants

The inclusion criteria included supratentorial hypertensive ICH patients who were 18 to 80 years of age, had a hemorrhage volume ≥ 25 mL, were admitted to the hospital within 24 h of ictus, had a Glasgow Coma Scale (GCS) score ≥ 5, and had a modified Rankin scale (mRS) score of 0 or 1 before hemorrhage. The exclusion criteria included concurrent head injury or cerebral tumor, multiple bleeding events, brain herniation at admission, hemorrhage due to aneurysm or arteriovenous malformation, and pregnancy. Detailed inclusion and exclusion criteria were presented in Additional file 1. All the chief surgeons who participated in this study had been involved in neurosurgery for more than 15 years and participated in training programs held by the Chinese PLA General Hospital. All the chief surgeons were proficient in all three surgical methods before recruiting patients.

### Randomization

Patients were randomly allocated to endoscopy group, aspiration group, or craniotomy group at a 1:1:1 ratio using a randomization system based on computer-generated number sequences after providing written informed consent. Complete randomization was used to minimize selection bias, and neither the neurosurgeons nor the patients were familiarized with the grouping beforehand. Randomization was completed within 24 h of hemorrhage, and patients were operated on within 12 h after randomization. The independent study statistician was blinded to the grouping information. Blinding of treating neurosurgeons and investigators was infeasible due to the nature of the surgery.

### Procedures

In contrast to the method used in previous studies, we used the open-source software 3D Slier (http://www.slicer.org/) to reconstruct and digitally measure the volume of hematoma since the traditional ABC/2 method usually overestimates hematoma volume, especially for irregular hematomas [18]. The process of hematoma 3D reconstruction was presented in Appendix 1 in Additional file 2. Accurate measurement of hematoma volume by computer could reduce estimation error and improve the comparability of data between participating centers. CT angiography (CTA) was performed when the underlying vascular pathology was suspected to be a source of bleeding. The indications for CTA included lobar hemorrhage in patients younger than 70 years, deep hemorrhage in patients younger than 45 years, and subarachnoid hemorrhage.

In the endoscopic surgery group, a bone flap approximately 2 cm in diameter was made according to the position of the hemorrhage. Surgical approaches included the middle frontal gyrus approach for basal ganglia hemorrhage, the parietooccipital approach for thalamic hemorrhage, and the subcortical approach for superficial lobar hemorrhage. Using a self-developed neuro-endoport (Chinese invention patent number: 201210066281.1), a transparent working channel was made to evacuate the hematoma under endoscopic surveillance [19]. Thrombolytic agents were not used after surgery. In the stereotactic aspiration group, the target point was set near the posterior edge of the hematoma on CT, which was the location at which the largest expansion of the hematoma occurred. Available commercial frameless neuro-navigation or free mobile-device-based navigation, rather than stereotactic head frame, was used to improve the accuracy of catheter puncture [20]. Initial hematoma aspiration was performed intraoperatively immediately after catheter insertion. After confirmation of the catheter by postoperative CT, urokinase (30,000 units every 12 h) was injected through the catheter to dissolve residual hematoma for 3–5 days [21]. The catheter was removed when there was no additional blood drained or when the catheter had been placed for 5 days. In the small-boneflap craniotomy group, the chief surgeon chose a suitable surgical approach based on the hemorrhage location and made a minimal boneflap to evacuate the hematoma as much as possible under microscope. Decompressive craniectomy was performed only when the brain tissue swelled significantly after hematoma evacuation. All patients were then transferred to the neurosurgical intensive care unit (NICU) until they were evaluated as stable enough for the general ward. We followed the guidelines for the management of spontaneous ICH from the American Heart Association and American Stroke Association, which enabled a standard schedule for monitoring patients’ blood pressure, airways, sedation, and pharmacological treatment [22].

### Outcomes

The primary outcome was the proportion of patients who achieved a favorable outcome, defined as a mRS score of 0–2 at 6 months after hemorrhage. Patients were adjusted for group differences in the main baseline covariates, such as sex ratio, age, preoperative GCS score, time from onset to surgery, and hematoma volume. Secondary outcomes included quality of life measured with the Barthel Index at the 6-month follow-up, mortality rate at one month, days of NICU stay, hospitalization time, intracranial infection rate, hospitalization expenses, hematoma clearance rate and operation time. The stroke-related pneumonia rate was also recorded as a secondary outcome. Patients were followed-up by investigators blinded to the treatment allocation through online video interviews and questionnaires 1 month and 6 months after treatment. A special case report form (see Appendix 2 in Additional file 2) was designed for data collection and outcome assessment. Adverse events were reported by the investigators throughout the study and were assessed by an independent supervisor (JSZ). An independent data safety and monitoring committee was appointed by the study sponsor.

### Statistical analysis

Our previous retrospective study indicated that an estimated 38% of patients in the endoscopy group would have an mRS score of 0–2, but only 17% of craniotomy patients would have the same outcome [19]. We estimated that 720 patients (240 patients in each treatment group) would provide 90% power and a type I error probability of 0.05 to detect an effect size of 12%, with a 10% dropout rate taken into consideration. Statistical analysis was performed on an “intention-to-treat” basis by treatment allocation. The primary outcome analysis was a simple categorical frequency comparison using the chi-square test for prognosis-based favorable or unfavorable outcomes based on the mRS at 6-month follow-up. The secondary outcome analysis consisted of one-way analysis of variance (ANOVA) for continuous variables and logistic regression analysis for age, gender, preoperative GCS score, hematoma location, hematoma volume, surgical method, and intracranial infection. A two-sided *P* < 0.05 was considered to indicate statistical significance.

## Results

### Clinical characteristics

**Fig. 1:**
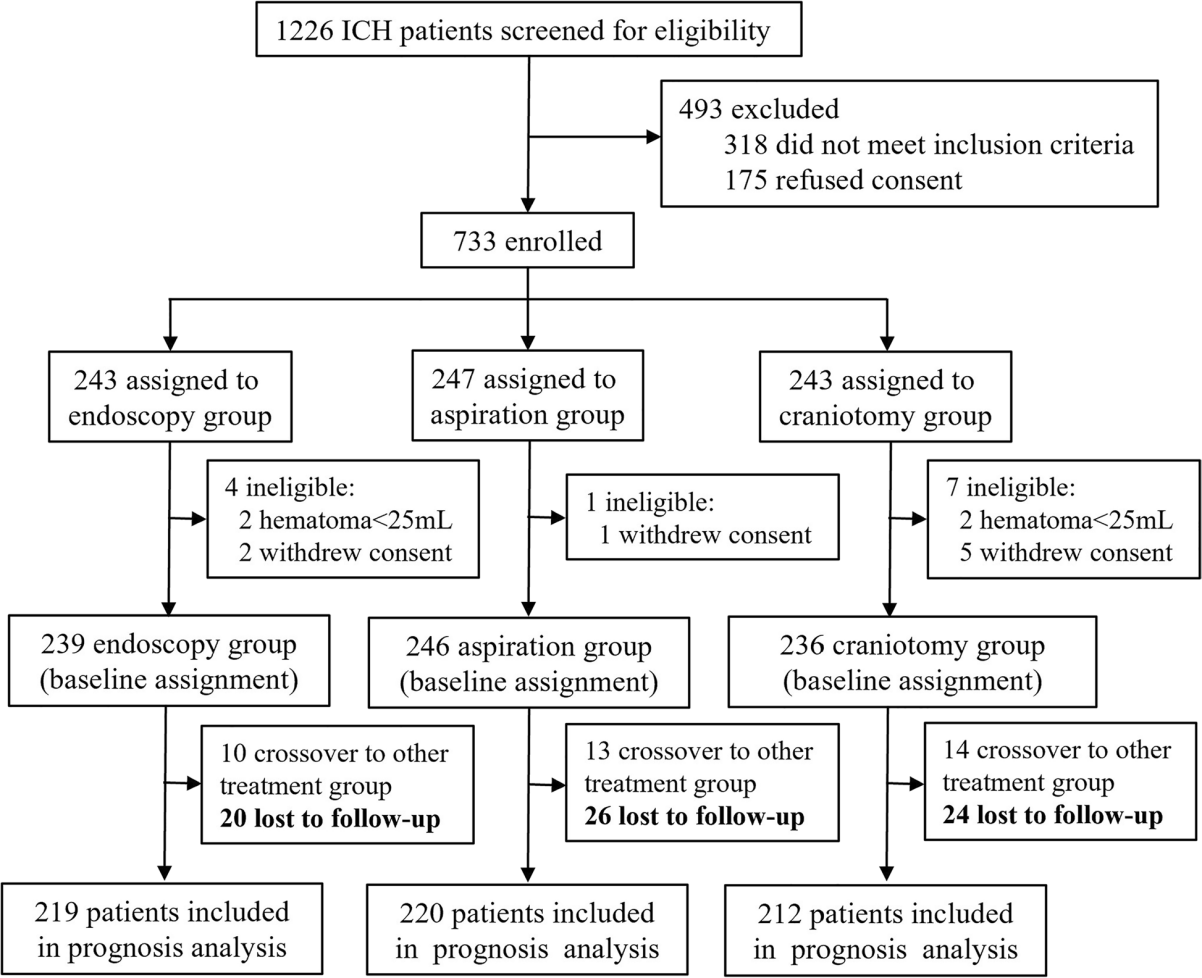
Trial profile

Between July 1, 2016, and June 30, 2022, 1226 ICH patients from 16 hospitals across China were screened for eligibility; 733 patients were randomly allocated to the three study groups. Among the 493 patients who were excluded from the trial, 318 (64.5%) did not meet the inclusion criteria, and 175 (35.5%) refused consent. Further 12 patients were excluded due to ineligible hematoma volume or consent withdrawal before treatment and were not included in the analysis. Finally, 721 patients (239 in the endoscopy group, 246 in the aspiration group, and 236 in the craniotomy group) received treatment and were included in the intention-to-treat analysis (Fig. 1). The demographic and baseline characteristics of patients were summarized in Table 1. The results showed that 497 (68.8%) of 721 patients were men and 225 (31.2%) were women (mean age 56.7 years, SD 11.3); 395 (54.8%) were left-sided hemorrhage and 326 (45.2%) were right-sided hemorrhage. For hemorrhage location, 515 (71.4%) were located mainly in the basal ganglia, 144 (20.0%) located in the lobar region, and 62 (8.6%) located in the thalamus. Forty-eight (20.1%) patients in the endoscopy group, 53 (21.5%) patients in the aspiration group, and 42 (17.8%) patients in the craniotomy group involved intraventricular hemorrhage (*P* = 0.598). The mean Graeb score of these patients were 2.5 (SD 0.8) in the endoscopy group, 2.7 (SD 1.0) in the aspiration group, and 2.6 (SD 0.9) in the craniotomy group (*P* = 0.264). On admission, the mean hematoma volume was 49.2 mL (SD 17.7, range 25.0–112.3), and the median GCS score was 9 (IQR 7–11). Medical conditions, past medical history, and the proportion of patients taking anticoagulants were similar among three groups. The preoperative characteristics of patients were balanced and comparable among three groups.

**Table 1:** Demographic and baseline characteristics of recruited patients

	**Endoscopy group (*n* = 239)**	**Aspiration group (*n* = 246)**	**Craniotomy group (*n* = 236)**
Sex			
Men	166 (69.5%)	170 (69.1%)	160 (67.8%)
Women	73 (30.5%)	76 (30.9%)	76 (32.2%)
Age (years)	56.6 (SD 11.0)	57.8 (SD 11.0)	55.8 (11.8)
GCS score	9.0 (SD 3.1)	9.4 (SD 3.2)	8.9 (SD 3.0)
Antiplatelet therapy	12 (5.0%)	22 (8.9%)	15 (6.4%)
BP at randomization			
Systolic (mmHg)	176.1 (SD 32.2)	172.5 (SD 32.1)	179.5 (SD 34.8)
Diastolic (mmHg)	100.0 (SD 19.4)	99.5 (SD 18.9)	101.3 (SD 22.7)
Past medical history			
Ischemic heart disease	12 (5.0%)	15 (6.1%)	14 (5.9%)
Diabetes mellitus	19 (7.9%)	21 (8.5%)	22 (9.3%)
Previous stroke	20 (8.4%)	18 (7.3%)	17 (7.2%)
Chronic kidney disease	6 (2.5%)	8 (3.3%)	5 (2.1%)
Time from ictus to surgery (h)	18 (IQR 6–25)	19 (IQR 6–29)	17 (IQR 6–26)
Side			
Left	133 (55.6%)	134 (54.5%)	128 (54.2%)
Right	106 (44.4%)	112 (45.5%)	108 (45.8%)
Hematoma location			
Basal ganglia	169 (70.7%)	177 (72.0%)	169 (71.6%)
Lobar	45 (18.8%)	50 (20.3%)	49 (20.8%)
Thalamus	25 (10.5%)	19 (7.7%)	18 (7.6%)
ICH volume (mL)	49.1 (SD 20.3)	48.5 (SD 14.9)	49.9 (SD 17.6)

*GCS* Glasgow Coma Scale, *BP* Blood pressure, *ICH* Intracerebral hemorrhage, *SD* Standard deviation

The mean hematoma clearance rate was 88.3% (SD 20.8) in the endoscopy group, 60.3% (SD 25.6) in the aspiration group, and 86.5% (SD 17.9) in the craniotomy group (*P* = 0.000). The time required for surgery was 1.0 h in the aspiration group, 2.0 h in the endoscopy group, and 3.5 h in the craniotomy group (*P* = 0.000). In terms of intraoperative blood loss, endoscopy (88 mL) and aspiration (38 mL) was less than that of craniotomy (268 mL) (*P* = 0.000). The percentage of patients receiving intraoperative blood transfusion was 8.4% (20/239) in the endoscopy group, 2.8% (7/246) in the aspiration group, and 24.6% (58/236) in the craniotomy group, with statistical significance (*P* = 0.000). Nearly one-third of patients in the craniotomy group (31.9%, 75/236) developed stroke-related pneumonia, which was higher than that in the endoscopy group (22.6%, 54/239) and the aspiration group (18.3%, 45/246), the difference was statistically significant (*P* = 0.002). The incidence of intracranial infection was 4.6% (11/239) in the endoscopy group, 6.9% (17/246) in the aspiration group, and 5.9% (14/236) in the craniotomy group, with no statistically significant difference (*P* = 0.553). The median time in NICU was 5 days in the endoscopy group, 4 days in the aspiration group, and 6 days in the craniotomy group (*P* = 0.042), though the median hospitalization time were similar among three groups (22 vs 23 vs 23, *P* = 0.083). The mean hospitalization expenses were ¥92,420 in endoscopy group, ¥77,351 in aspiration group, and ¥100,947 in craniotomy group (*P* = 0.000). Stereotactic aspiration was the most economical surgical method. The general clinical results were summarized in Table 2.

**Table 2:** Treatment variables

Variables	Endoscopy group (*n* = 239)	Aspiration group (*n* = 246)	Craniotomy group (*n* = 236)	*P* value
Operation time (h)	2.0 (0.7)	1.0 (0.7)	3.5 (1.2)	0.000
Intraoperative blood loss (mL)	88 (84)	38 (71)	268 (228)	0.000
Hematoma clearance rate (%)	88.3 (20.8)	60.3 (25.6)	86.5 (17.9)	0.000
Stroke-related pneumonia	54 (22.6%)	45 (18.3%)	75 (31.9%)	0.002
Intracranial infection	11 (4.6%)	17 (6.9%)	14 (5.9%)	0.553
Time in NICU (days)	5 (3–9)	4 (1–8)	6 (3–9)	0.042
Hospitalization time	22 (14–37)	23 (14–35)	23 (15–33)	0.803
Hospitalization expenses (¥)	92,420 (50,330)	77,351 (62,511)	100,947 (60,749)	0.000

Data are expressed as median (IQR), mean (SD), or n/N (%)

*NICU *Neurosurgical intensive care unit, *IQR *Interquartile range, *SD *Standard deviation

### Functional outcome

**Fig. 2:**
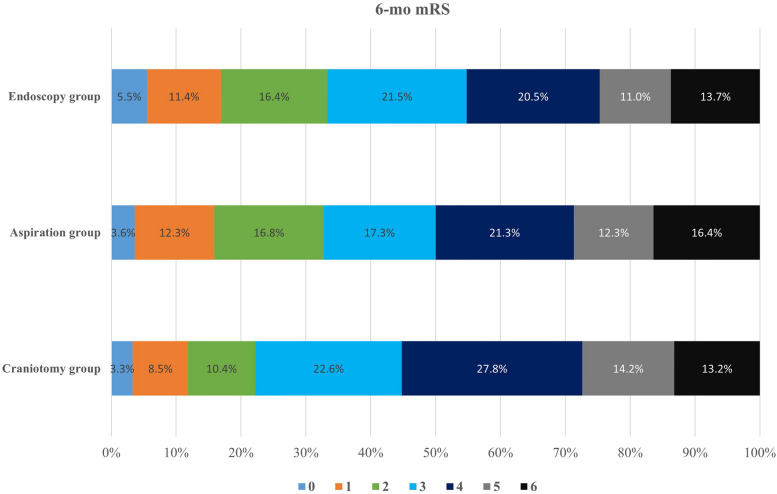
Modified Rankin Scale at 6-month follow-up

Ten patients in the endoscopy group, 13 patients in the aspiration group, and 14 patients in the craniotomy group crossover to other treatment groups. The reasons for cross-over contained refusal by relatives (18), neurological deterioration (8), relative contradiction for craniotomy or endoscopic surgery due to other major clinical event (6), and reason not recorded (5). The statistical analysis was based on initial grouping. At 6 months after ictus, 70 patients were lost to follow-up. Of the 651 patients with available mRS scores and BI scores at 6-month follow-up, 73 (33.3%) of 219 patients in the endoscopy group, 72 (32.7%) of 220 patients in the aspiration group, and 47 (22.2%) of 212 patients in the craniotomy group achieved a mRS score of 0–2. The long-term prognosis outcome was presented in Table 3. The proportion of patients with favorable outcome in the endoscopy group and the aspiration group was greater than that in the craniotomy group (Fig. 2, *P* = 0.017). Compared with small-boneflap craniotomy, the prognostic advantage of endoscopic surgery was 11.1% and that of frameless navigated aspiration was 10.5%. The 6-month mortality was 13.7% (30/219) in the endoscopy group, 15.0% (33/220) in the aspiration group, and 16.5% (35/212) in the craniotomy group. The difference was insignificant (*P* = 0.717). The median Barthel Index of alive patients at 6-month follow-up was 75 (IQR 45–95) in the endoscopy group, 75 (IQR 55–95) in the aspiration group, and 70 (IQR 40–90) in the craniotomy group. Patients in the endoscopy group and the aspiration group got better quality of lives than those in the craniotomy group (*P* = 0.019).

**Table 3:** Follow-up results at 6 months after hemorrhage

Variables	Endoscopy group (*n* = 219)	Aspiration group (*n* = 220)	Craniotomy group (*n* = 212)	*P* value
Outcome				
Favorable (mRS 0–2)	73 (33.3%)	72 (32.7%)	47(22.2%)	0.017
Unfavorable (mRS 3–6)	146 (66.7%)	148 (67.3%)	165 (77.8%)	
Mortality				
Dead	30 (13.7%)	36 (16.4%)	28 (13.2%)	0.601
Alive	189 (86.3%)	184 (83.6%)	184 (86.8%)	
Barthel Index	75 (45–95)	75 (55–95)	70 (40–90)	0.019

Data are median (IQR), or n/N (%)

*IQR* Interquartile range

Age, gender, preoperative GCS score, hematoma location, hematoma volume, surgical method, and intracranial infection were included in the non-conditional logistic regression analysis to identify any variable that correlated with patients’ functional outcome. Young age, better preoperative GCS score, small hematoma volume, lobar hematoma location, endoscopic surgery or stereotactic aspiration, and no intracranial infection were associated with higher odds of favorable outcome (6-month mRS 0–2). The odds ratios for age, preoperative GCS score, hematoma volume, endoscopic surgery, stereotactic aspiration, and intracranial infection were 1.046 (*P* = 0.000), 0.848 (*P* = 0.00), 6.159 (*P* = 0.000), 1.034 (*P* = 0.000), 0.383 (*P* = 0.000), 0.453 (*P* = 0.003), and 6.518 (*P* = 0.004), respectively (Table S1 in Additional file 2). Further analysis showed that for patients with supratentorial deep hemorrhages (basal ganglia hemorrhages and thalamus hemorrhages), the probability of achieving a favorable outcome was 30.3% (54/178) for endoscopic surgery, 28.4% (50/176) for stereotactic aspiration, and 14.8% (25/169) for craniotomy. The results had a significant statistical difference (*P* = 0.001). For patients with supratentorial lobar hemorrhages, the probability of achieving a favorable outcome was 46.3% (19/41) for endoscopic surgery, 50.0% (22/44) for stereotactic aspiration, and 51.2% (22/43) for craniotomy. The prognosis of craniotomy surgery was slightly better, but the difference was not statistically significant (*P* = 0.900). The probability of achieving a favorable outcome was similar between left-sided ICH (30.0%, 88/293) and right-sided ICH (29.6%, 106/358) (*P* = 0.542). Among the 125 followed-up patients with a GCS score of greater than or equal to 13 before surgery, 54.8% (23/42) in the endoscopic surgery, 53.5% (23/43) in the stereotactic aspiration, and 42.5% (17/40) in the craniotomy achieved a favorable outcome. The difference did not reach statistical significance (*P* = 0.477).

Specified safety event (such as severe rebleeding or sudden brain herniation) rates did not reach the predetermined review threshold throughout the trial. In the endoscopy group, 9 of 239 (3.8%) patients experienced rebleeding after surgery, and 6 patients underwent reoperation. Furthermore, one patient received external ventricular drainage because of hydrocephalus after hemorrhage, one patient underwent third ventriculostomy and fistulation of the entrapped temporal horn, and one patient suffered from bleeding at tracheotomy. In the aspiration group, 15 of 246 (6.1%) patients experienced rebleeding after surgery, 4 patients underwent a second-time stereotactic aspiration, and 2 patients underwent craniotomy. Two patients received external ventricular drainage because of hydrocephalus after hemorrhage. In the craniotomy group, 12 of 236 (5.1%) patients experienced rebleeding after surgery, and 7 patients underwent craniotomy again. Three patients received external ventricular drainage because of hydrocephalus. Decompressive craniotomy was performed in only 8 patients (3.4%) in the craniotomy group. The percentage of patients suffering from rebleeding did not reach statistical significance. At the 1-month follow-up, the mortality rates were similar among the three groups (17/239, 7.1% vs 20/246, 8.1% vs 19/236, 8.1%, *P* = 0.898). Until 6-month follow-up, 11 patients in the endoscopy group, 24 patients in the aspiration group, and 20 patients in the craniotomy group develop hydrocephalus that required shunt surgery. The difference was insignificant (*P* = 0.052).

## Discussion

As the deadliest form of acute stroke, ICH has been a great public health threat with persistently high mortality in recent decades [4, 7, 23]. Novel treatments and improved application of established approaches are needed for ICH [22]. The usefulness of craniotomy for most patients with spontaneous supratentorial ICH of moderate severity remains uncertain [10, 11, 24–27]. For large supratentorial ICH accompanied with clinical deterioration and significant midline shift, decompressive craniectomy with or without hematoma evacuation is considered a lifesaving procedure [24, 28]. A variety of possible reasons have been proposed for why craniotomy fails to improve ICH outcomes, including operation beyond the optimal treatment time, insufficient sample size, incomplete hematoma removal, and relatively high cross rate between groups. The damage to normal brain tissue caused by surgery and its impact on the postoperative recovery of patients have not been thoroughly studied.

There is still a long way to go in the clinical research of ICH. The STICH study compared early surgery with initial conservative treatment for patients with ICH and the results showed that early surgery had no benefit compared with initial conservative treatment. The STICH II study demonstrated that early surgery might have a small but clinically relevant survival advantage for patients with superficial ICH. The MISTIE III study found that for moderate to large ICH, minimally invasive catheter evacuation followed by thrombolysis did not improve the proportion of patients who achieved a good response 365 days after ICH compared with standard medical treatment. STICH and STICH II used the 8 point extended Glasgow Outcome Scale (GOS) and MISTIE III used the modified Rankin Scale (mRS). The proportion of patients with favorable outcome was 26% and 24% in STICH, 41% and 38% in STICH II, and 45% and 41% in MISTIE III [10, 11, 13]. Recently, minimally invasive surgeries have the appeal of relieving hematoma volume, reducing perihematomal edema, and minimizing disruption of healthy brain tissue. Minimally invasive surgeries were reported to be helpful for reducing mortality and improving functional outcomes compared with conventional craniotomy or standard medical treatment [12–16] [29–32]. Studies comparing minimally invasive surgery with craniotomy were all small sample RCTs, retrospective data or meta-analysis. In this multicenter large-scale RCT, endoscopic surgery and stereotactic aspiration improved functional outcomes compared with craniotomy in patients with supratentorial ICH. At 6-month follow-up, 33.3% patients in the endoscopy group and 32.7% patients in the aspiration group achieved a favorable outcome (mRS score 0–2), which was significantly greater than that (22.2%) in the craniotomy group. Patients who underwent minimally invasive surgeries also got better quality of lives. The prognosis advantage of endoscopic surgery and stereotactic aspiration over craniotomy was mainly reflected in patients with deep hemorrhages. For patients with superficial lobar hemorrhages, there was no statistically significant difference in the prognostic outcomes among the three surgical methods. The proportion of patients with lobar hemorrhage in our study was relatively small, and the results warranted further investigation.

There was no significant difference in mortality at 6 months after hemorrhage. Previous studies have shown that for patients with supratentorial ICH and intraventricular hemorrhage, endoscopic surgery or stereotactic aspiration with or without thrombolytic use reduces mortality compared with craniotomy [12, 13, 33, 34]. The reasons for the similar 6-month mortality in three groups might be that the overall mortality of the enrolled patients was relatively low, some patients were lost to follow-up or crossed over, and our 6-month follow-up did not record the specific cause of death. Furthermore, our results provide important health economic evidence that stereotactic aspiration is the most economical surgical method. Our study provides important new evidence for minimally invasive treatment of spontaneous hypertensive ICH. Apart from deep hemorrhage location, occurrence of intraventricular hemorrhage (IVH) was also was an important predictor of poor outcome. Post-hemorrhagic hydrocephalus develops in 30%-50% of patients with IVH [35]. Endoscopic surgery was reported to not only improve the prognosis but also reduce shunt dependence after IVH [36]. Patients with pure IVH or IVH volume exceeding 50% of the total hematoma volume were not included in our study, therefore, the proportion of IVH in our series was low and the difference did not reach statistical significance.

A follow-up rate of 90.3% was achieved, and the crossover rate was controlled to a relatively low level. The MISICH trial used the intention-to-treat principle; that is, the analysis of outcomes was based on the patients’ initial randomization results rather than the final treatment that the patient received to avoid the influence of human factors that may influence the results. All three surgical methods were safe regarding serious rebleeding and intracranial infection, and no obvious procedural risk was reported in all groups. The potential impact of the timing of surgery for ICH on outcome remains controversial, and the best therapeutic time window is unknown [32, 37]. STICH I and STICH II did not identify an early time effect [10, 11]. In MISICH, surgery was performed at 6-48 h since hematoma expansion, an indication of unfavorable outcome, occurred in nearly one third patients and mostly occurred within 6 h after haemorrhage [38, 39]. No surgery was performed within 6 h in our study. Further research evaluating the best surgery timing was warranted. The relationship between hematoma clearance rate and patient prognosis remains unclear. The hematoma clearance rate of stereotactic aspiration was lower than that of craniotomy, but the recovery of neurological function was better, indicating that within a certain range, increasing hematoma clearance rate could improve prognosis; however, when the hematoma is cleared to a certain extent, further removal of hematoma could not provide additional benefit. The cutoff clearance rate is still unknown and it is generally recommended to remove the hematoma until the residual hematoma volume is less than 15 ml [11, 13, 22, 24]. We conducted subgroup analysis based on whether the residual hematoma volume was greater than 15 ml. The results showed that 31.9% (151/475) of patients with residual hematoma less than 15 ml achieved a favorable outcome (mRS 0–2) versus 23.0% (41/178) of patients with residual hematoma more than or equal to 15 ml achieved a favorable outcome (*P* = 0.017).

This study has several limitations. First, insufficient power might be an issue due to the moderate size of the intention-to-treat analysis, while dichotomous variables (favorable or unfavorable) usually require a large sample size. Second, the inclusion criteria might exclude some patients with unfavorable prognosis and whether patients with GCS scores of 5 suitable for enrollment left room for discussion. Third, the number of patients recruited from participating centers differed, potentially resulting in regional biases, which might be a confounding factor.

## Conclusions

This prospective multicenter RCT demonstrated that, compared with small-bone flap craniotomy, endoscopic surgery and frameless navigated aspiration significantly improved the functional outcomes and quality of life of patients with moderate to large supratentorial hypertensive ICH, especially deep hemorrhages. Frameless navigated aspiration and endoscopic surgery were also more cost-effective compared with small-bone flap craniotomy. More powered clinical data are warranted to compare endoscopic surgery with stereotactic aspiration.

### Supplementary Information

Additional file 1: Trial protocol.

Additional file 2: Appendix 1. Digital Hematoma Volume Measurement with 3D Slicer. Appendix 2. MISICH Case Report Form. Table S1. Logistic regression for unfavorable outcome.

## Data Availability

The data that support the findings of this study are available from the corresponding author upon reasonable request.

## Abbreviations

ANOVA: One-way analysis of variance
GCS: Glasgow Coma Scale
ICH: Intracerebral hemorrhage
IQR: Interquartile range
IVH: Intraventricular hemorrhage
MISICH: Minimally invasive surgeries for spontaneous hypertensive intracerebral hemorrhage
mRS: Modified Rankin Scale
NICU: Neurosurgical intensive care unit
SD: Standard deviation
