# Supplementary material for: Minimally invasive surgeries for spontaneous hypertensive intracerebral hemorrhage (MISICH): a multicenter randomized controlled trial
Source: BMC Med. 2024 Jun 13;22:244. doi: 10.1186/s12916-024-03468-y (PMC11170771; doi:10.1186/s12916-024-03468-y)
Supplement: Supplementary file 1 — Additional file 1: Trial protocol. [file 12916_2024_3468_MOESM1_ESM.docx]

**Minimally Invasive Surgeries for Hypertensive Intracerebral Hemorrhage (MISICH): Study Protocol**

**Department of Neurosurgery**

**Chinese PLA General Hospital**

**Background**

Hypertensive intracerebral hemorrhage (HICH) is a crucially important neurological emergency characterized by high fatality and disability rate. HICH accounts for about 70% of all intracerebral hemorrhage (ICH) cases. It affects about 4 million people worldwide each year and the median mortality at 1 month is 40%. Many survivors remain severely disabled, and only 12% of the survivors could live independently with minor handicap after 6 months, posing a huge burden on the society and families.

Theoretically, surgery has the potential to improve neurological recovery after ICH since early removal of the hematoma might reduce nervous tissue damage, possibly by relieving local ischemia or removal of noxious chemicals. However, the effectiveness of surgery (mainly refers to craniotomy) in the treatment of ICH remains controversial. Several prospective randomized controlled trials (RCTs) have been undertaken during the past 4 decades, but results of most individual trials failed to demonstrate improvement in outcome in surgically treated patients. Results of the STICH trial indicates that patients with spontaneous supratentorial ICH in neurosurgical units show no overall benefit from early surgery when compared with initial conservative treatment. The STICH II trial, a study based on subgroup analysis of the STICH trial, confirms that early surgery does not increase the rate of death or disability at 6 months and might have a small but clinically significant survival advantage for patients with spontaneous superficial ICH.

Minimally invasive surgery generally refers to the concept of creating minimal trauma to normal brain tissue during the process of removing hematoma. This stands in distinction from the open craniotomy in which a large bone flap is created, the brain is exposed, retracted, and manipulated to inspect the site of bleeding and suction blood from multiple areas. Two main types of minimally invasive surgery have been attempted for hematoma removal: endoscopic evacuation and stereotactic aspiration. In endoscopic evacuation, a small boneflap is created, and an endoscope is inserted through normal brain tissue into the hematoma with the help of an introducer. Suction and irrigation are applied to remove the hematoma. Auer reported the first randomized trial of endoscopic-guided hemorrhage evacuation with a sample size of 100. Results indicated that endoscopic surgical evacuation offers promise to maximize hematoma evacuation while minimizing damage to normal tissue. Stereotactic aspiration involves using image guidance to place a catheter into the main body of the hematoma and aspirate blood. A catheter is left in the body of the hematoma, and during the course of several days, repeated small doses of thrombolytic agents is instilled via the catheter into the brain to clear the left hematoma slowly. Recently, several studies exploring the efficacy of minimally invasive surgery compared with craniotomy or medical treatment have been carried out, but none of them provided sufficient evidence regarding the choice of treatment. Until now, no results of largescale RCTs comparing the efficacy of endoscopic evacuation, stereotactic aspiration and craniotomy in patients with ICH has been reported. Here we designed a multicenter, randomized, controlled trial to investigate whether minimally invasive hematoma evacuation with endoscopic or stereotactic aspiration will improve functional outcome in patients with supratentorial ICH compared with small boneflap craniotomy.

**Methods/design**

**Objectives**

This trial primarily aims to investigate whether and to what extent endoscopic evacuation and stereotactic aspiration could improve the outcome of supratentorial HICH compared with small boneflap craniotomy. This trial will also help to better define the indications for different surgical methods and help to determine the best surgical method for treatment of supratentorial HICH. Another objective is to evaluate the separate effects of different surgical treatments on patients’ independency and activities of daily living.

**Study design**

This is a multicenter, randomized, controlled, open-label, sequentially designed non-profit study (ClinicalTrials.gov ID: NCT02811614) involving 16 neurosurgical centers. Patients with newly diagnosed supratentorial HICH according with our inclusion criteria will be enrolled in this study. A follow-up period of 6 months will be sufficient enough to show outcome and prognosis of surgical treatment. A flowchart of this study is shown in Figure 1.


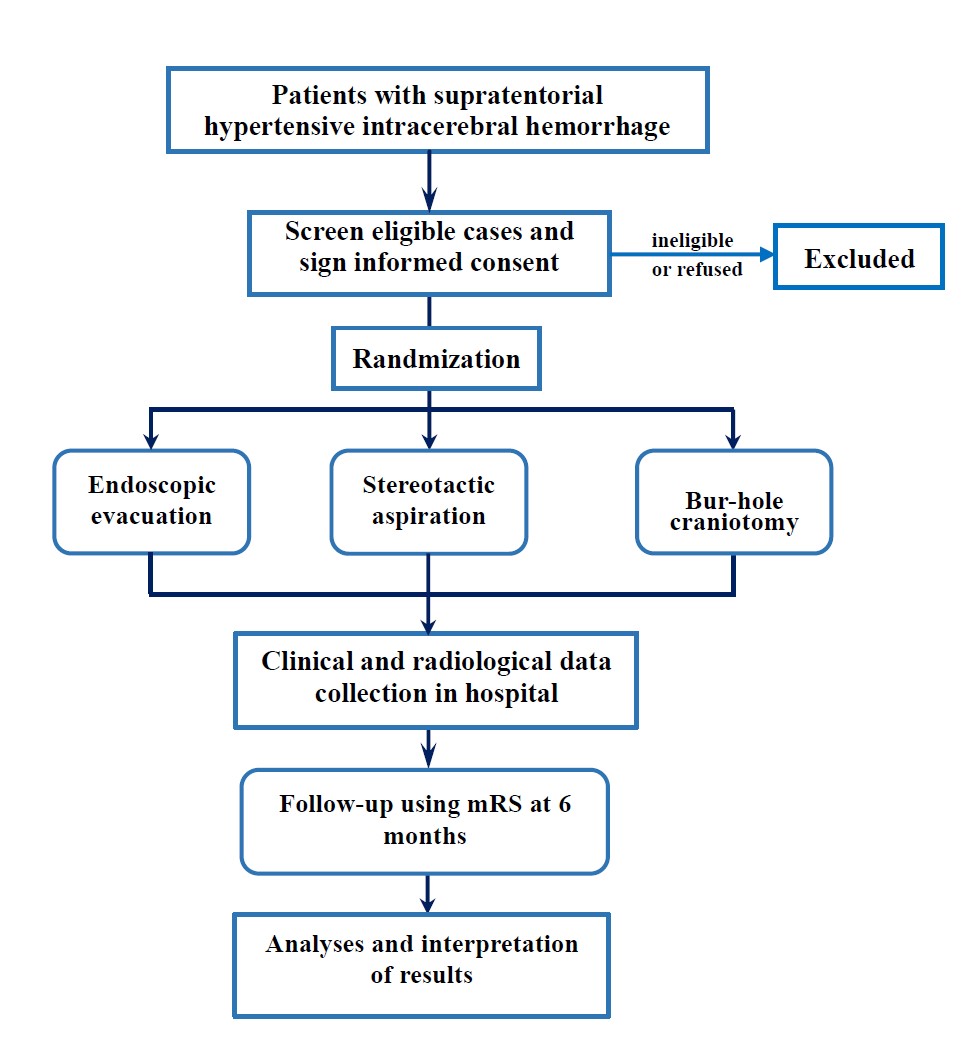


Figure 1 Flow chart of the study design

The trial will be conducted in the departments of neurosurgery at 16 Chinese hospitals: (1) Chinese PLA General Hospital, Beijing; (2) Jingzhou Central Hospital, Hubei province; (3) Wuhan No.1 Hospital, Hubei province; (4) Jinzhou Central Hospital, Liaoning province; (5) Yichang Central People’s Hospital, Hubei province; (6) Hainan Hospital of Chinese PLA General Hospital, Hainan province; (7) Liaoning Thrombus Hospital, Liaoning province; (8) Jiangmen Central Hospital, Guangdong province; (9) People’s Hospital of Sanshui District. Foshan, Guangdong province; (10) Minzu Hospital of Guangxi Medical University, Guangxi province; (11) The Second Hospital of Jilin University, Jilin province; (12) Jilin Province People’s Hospital, Jilin province; (13) Siping Central People’s Hospital, Jilin province; (14) Tangdu Hospital of Air Force Military Medical University, Shanxi province; (15) The Third Affiliated Hospital of Inner Mongolia Medical University, Inner Mongolia; (16) The First Affiliated Hospital of Xinjiang Medical University, Xinjiang province. All neurosurgeons taking part in the MISICH study have been trained by an annually held national continuing education program by Chinese PLA General Hospital about endoscopic evacuation, stereotactic aspiration, and small boneflap craniotomy. These three surgical techniques have been routinely used in all centers prior to the start of trial.

**Eligibility criteria**

***Inclusion criteria***

1. Men and women aged 18-80 years old

2. Present with supratentorial HICH confirmed by brain computed tomography (CT) scan, hematoma volume ≥25mL

3. Glasgow Coma Scale (GCS) score ≥5

4. Patients are admitted to the above-mentioned hospitals within 24 hours of ictus

5. Patients or their dependents (when patients in coma) provide written consent.

***Exclusion criteria***

1. The hemorrhage is caused by cerebral tumor, coagulopathy, aneurysm or arteriovenous malformation

2. With concurrent head injury or history of head injury

3. There are multiple hemorrhagic lesions

4. Have severe dementia or disability

5. Patients already have indications of terminal brain hernia

6. Patients with concomitant diseases that will affect their life expectancy

7. Hematoma mainly located in ventricular system

8. Pregnant woman.

**Sample size and randomization**

***Sample size***

It was reported that the rate of unfavorable outcome of patients receiving craniotomy with a supratentorial hematoma volume >30 ml was 49% to 67% and minimally invasive surgeries might decrease the rate of unfavorable outcome by about 8% to 22% compared with craniotomy. Our recent retrospective study showed that endoscopic surgery lowered the risk of unfavorable outcome in patients with supratentorial HICH by nearly 28% in comparison with craniotomy. We assume that the endoscopic surgery and stereotactic aspiration could reduce the risk of unfavorable outcome from 33% to 21%. A sample size of 720 (240 in each group) would be required to show a 12% benefit from minimally invasive surgery with 90% power and a type I error probability of .05 with a 10% dropout rate taken into consideration.

***Randomization***

To minimize selection bias and accidental bias, Patients were randomly allocated to endoscopic evacuation group, aspiration group, or small boneflap craniotomy group in 1:1:1 ratio using a central randomization system based on computer-generated number sequence after a written informed consent was signed. Neither the neurosurgeons nor the patients could know the grouping beforehand. Randomization must take place within 24 hours of ictus and patients will be operated on within 12 hours of randomization. During the randomization process the neurosurgeon is informed of the treatment group the patient is allocated to and should record this. Best medical treatment must begin as soon as possible and continue throughout follow-up in all patients.

**Surgical procedures**

***Endoscopic evacuation***

In this intervention group, patients will receive endoscopic surgery under general anesthesia. A 4-5cm skin incision is made according to the position of hematoma on brain CT scan. The approaches include the middle frontal gyrus approach for anterior basal ganglia hemorrhages that are not elongated but rather are more spherical. A parietooccipital small boneflap is created to treat posterior basal ganglia and thalamic hemorrhages. In cases involving superficial lobar hemorrhages, use a small boneflap directly at the location where the lesion comes closest to the surface. Then make a bone flap about 2cm in diameter and incise the dura mater in a cruciate fashion. Use a new-developed endoscopic introducer (China National Invention Patent: 201210066281.1) to make an appropriate working channel for the endoscope. First, impale the blunt puncturing lever in a predetermined position and then pull out the inner core. Second, suck with a syringe to determine whether the puncture lever has been in the right place. After confirmation, place the transparent cannula along the puncture lever. Through the space made by the transparent introducer, we can carefully evacuate intracerebral hematoma under the surveillance of endoscope. Plasminogen activator (urokinase) will not be injected after endoscopic surgery.

***Stereotactic aspiration***

In the aspiration group, the target point was set at near the posterior edge of the hematoma chosen on CT scan image with the largest expansion of hematoma. First, the patient undergoes a brain CT scan with slice thickness <5mm and the CT image data in Digital Imaging and Communications in Medicine (DICOM) format will be collected. Available commercial neuro-navigation or free mobile-device-based navigation was recommended in all centers to improve accuracy of catheter puncture. The target point is chosen on the CT scan with the largest expansion of the hematoma and special attention was paid to stay away from important cortex function areas. Hematoma aspiration was done with a 10 mL syringe until first resistance. After confirmation of catheter by postoperative CT, urokinase of 30,000 units every 12h was injected through the catheter to dissolve residue hematoma for 3-5 days. Catheter was removed when there was no more blood drained or the catheter had been placed for up to 5 days.

***Small boneflap Craniotomy***

Patients in this group will receive hematoma evacuation by small boneflap craniotomy under general anesthesia. Well-trained neurosurgeons will decide the surgical approach according to hematoma location and size on CT scan. To minimize the damage to normal brain tissue, operative incision should be made as small as possible. After removing the bone and incise the dura mater, cut open the cortex to reach the hematoma on the basis of protecting functional cortex areas and blood vessels. The hematoma will be evacuated as much as possible with the help of operation microscope. After careful hemostasis, the blood pressure should be moderately elevated to confirm that there has been no bleeding site. Only when the brain tissue swells significantly after hematoma evacuation, the bone flap should be expanded and abandoned. When necessary, a catheter would be placed in the hematoma cavity though urokinase is not usually injected.

**Concomitant care and interventions**

All patients will be cared for in a neurosurgical intensive care unit (NICU) until they are considered stable enough to move to an intermediate care or general unit. Neurological status will be monitored in the NICU by GCS and hourly neurological evaluation, which includes vital signs, level of consciousness and limb muscular strength.

**Outcomes measurements**

The primary outcome of this study is the degree of disability estimated using the modified Rankin Scale (mRS) 6 months after ictus. An unfavorable outcome is defined as death or dependency with a mRS of 3-6. Living patients’ independence in activities of daily lives and quality of life will be a highlight of this study. We will also measure patients’ quality of life and their performance in activities of daily living using the Barthel Index at 6-months follow-up. Secondary outcomes also include: hematoma clearance rate evaluated at 72h after surgery, operation time, intraoperative blood loss, postoperative GCS score 7 days after surgery, rebleeding rate, days of NICU stay, intracranial infection rate during hospitalization, 1 month mortality, 3 months mortality, hospitalization expenses, and Barthel Index at 6 months.

**Data collection and follow-up**

A special case report form (CRF) with detailed record rules has been developed in order to ensure accurate data collection. Patients’ personal data are made anonymous and numbered by the system. A list of patient names and study numbers is kept in a separate file to ensure that patients’ confidentiality is maintained. Demographic, clinical and neurological data are recorded at the time of enrolment and throughout follow-ups. The filled CRFs should be sent to trial secretary expeditiously.

Copies of the CT scans at enrollment, 1 day, 3 days and 7 days after surgery will be sent to the trial secretary together with the CRFs. All CT data should be in DICOM compatible format. DICOM images are sent anonymously with only patient identifier visible. The images will be analyzed by trained readers blinded to treatment group and patient identifier. In the past studies, hematoma volumes are calculated almost all by the ABC/2 formula. However, there’s significant estimation error using the ABC/2 formula to calculate hematoma volume, especially in large irregular hematomas. In this trial, we will use a precise and free method, the software 3D Slicer to measure both preoperative and postoperative hematoma volumes (Figure 2).


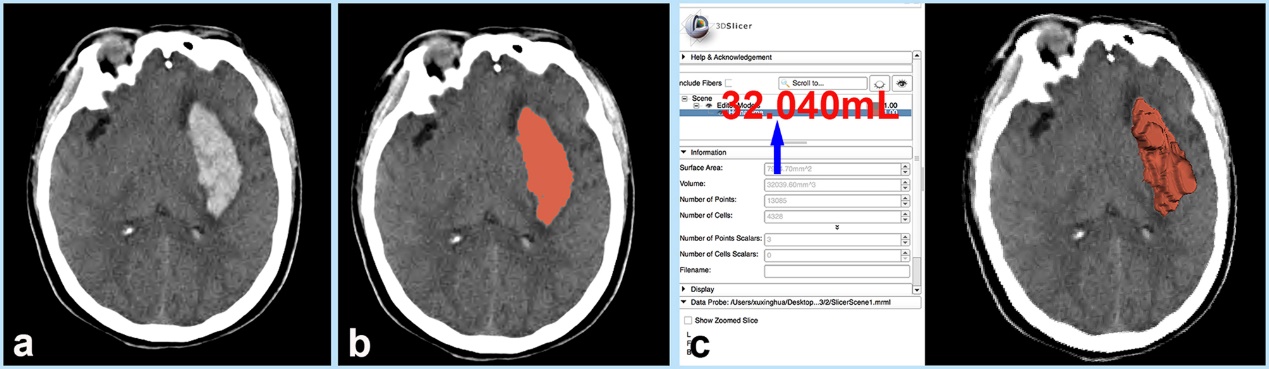


Figure 2 Hematoma volume measurement with software 3D Slicer. a. preoperative brain CT scan confirmed left external capsule hemorrhage; b. automatic depiction of the hematoma using threshold effect; c. 3D reconstruction and volume measurement of hematoma.

Follow-up data will be collected at 1 month, 3months, and 6 months after onset. All patients are encouraged to make a regular return visit to the hospital. Patients who cannot make a regular return visit will be followed-up through telephone or postal questionnaires. Our goal is to achieve at least 90% follow-up by means of full cooperation of all the investigators.

**Statistical analysis**

Statistical analysis will be on a “intention to treat” basis. Measurement data will be described using mean and standard deviation or median and interquartile range while count data will be described using indexes such as percentage, ratio and relative risk. The primary analysis will be a simple categorical frequency comparison using the Chi-squared test for prognosis based favorable and unfavorable outcomes at 6 months. The continuous variables will be analyzed by One-way ANOVA. Logistic regression analysis will be undertaken for age, gender, hematoma volume, preoperative GCS, surgical method, and intracranial infection. All statistical analyses will be performed using SPSS statistics 26.0 (IBM Corp. USA). A value of two side P <.05 was considered statistically significant.

**Data monitoring and quality control**

The data monitoring committee considers data from interim analyses and reports to the Trial Steering Committee. Interim analyses are strictly confidential and the committee will only recommend stopping the trial early if one or other treatment show an advantage at a very high significance level. The coordinating center (Chinese PLA General Hospital, Beijing) is responsible for maintaining computerized databases containing all data related to the trial, the quality of computerized information, conducting statistical analyses, preparing reports for the data monitoring committee, and all correspondence in relation to the trial. To better control the quality of this study, all neurosurgeons performing the operation have got a short-term centralism training before the trial begins.
