# Supplementary material for: Minimally invasive surgeries for spontaneous hypertensive intracerebral hemorrhage (MISICH): a multicenter randomized controlled trial
Source: BMC Med. 2024 Jun 13;22:244. doi: 10.1186/s12916-024-03468-y (PMC11170771; doi:10.1186/s12916-024-03468-y)
Supplement: Supplementary file 2 — Additional file 2: Appendix 1. Digital Hematoma Volume Measurement with 3D Slicer. Appendix 2. MISICH Case Report Form. Table S1. Logistic regression for unfavorable outcome. [file 12916_2024_3468_MOESM2_ESM.docx]

**Additional File 2**

**Supplemental Online Content**

**Appendix 1.** Digital Hematoma Volume Measurement with 3D Slicer

**Appendix 2.** MISICH Case Report Form

**Appendix 3.** Logistic regression for unfavorable outcome

**Appendix 1.** Digital Hematoma Volume Measurement with 3D Slicer

Intracerebral hematoma (ICH) is a significant cause of death and disability worldwide^1^. The volume of ICH has been validated to be an important independent predictor for prognosis. The ABC/2 formula has been widely used for bedside estimation of hematoma volume,^5^ assuming that hematoma shape is idealized ellipsoid. Clinically, hematoma shape is often irregular rather than ellipsoid. It is therefore reasonable to assume that the ABC/2 formula has a certain range of error, which, theoretically, will be larger in irregular or larger hematomas.

3D Slicer is a free open source software platform for biomedical research (<http://www.slicer.org>). It is similar to a radiology workstation but is free and not tied to specific hardware. 3D Slicer not only supports versatile visualizations but also provides advanced function, such as automated segmentation and registration for various applications. The latest stable release is version 5.6.0.

All roads lead to Rome. There are more than ten methods for 3D Slicer reconstruction and calculation of hematoma volume, but the basic principle is based on the difference between the CT value of hematoma on brain CT image and the CT value of surrounding normal brain tissue. The CT value of normal brain tissue is generally 25-45 Hounsfield unit (Hu), and the CT value of cerebral hemorrhage hematoma is generally 60-80 Hu, which will gradually decrease with the absorption of hematoma. Here we only introduce one method.

First, import brain CT data in DICOM format into 3D Slicer software. Enter the “Segment Editor” module, click “Add” to add a new segment, double-click the color module to adjust the color to red, and then select “Threshold” in the Effects tool block to set the threshold range from 50Hu to 100Hu. You can also drag the slider to adjust the threshold until the hematoma is well separated from the surrounding normal brain tissue, and then click “Apply”. Select “Islands” tool, select “Keep selected islands”, and then click “Apply” to remove the parts around the skull that are abnormally stained due to partial volume effect. Click “Show 3D” tool to observe and adjust in the 3D window. Then click “Segmentations”, select “Export” “Models”, and then click “Export”. At this time, the three-dimensional model of hematoma is rebuilt, click “Models” module and select the model just reconstructed, then click “Information” to see the hematoma volume and other information (eFigure 1 to eFigure 5).


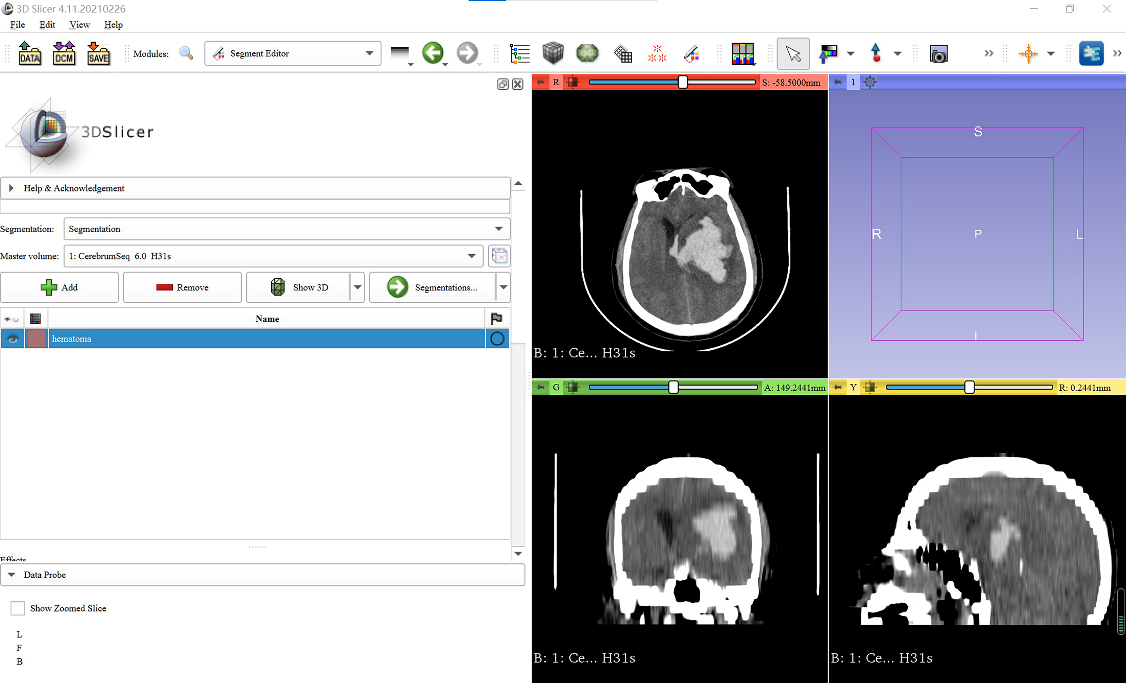


eFigure 1 Create new


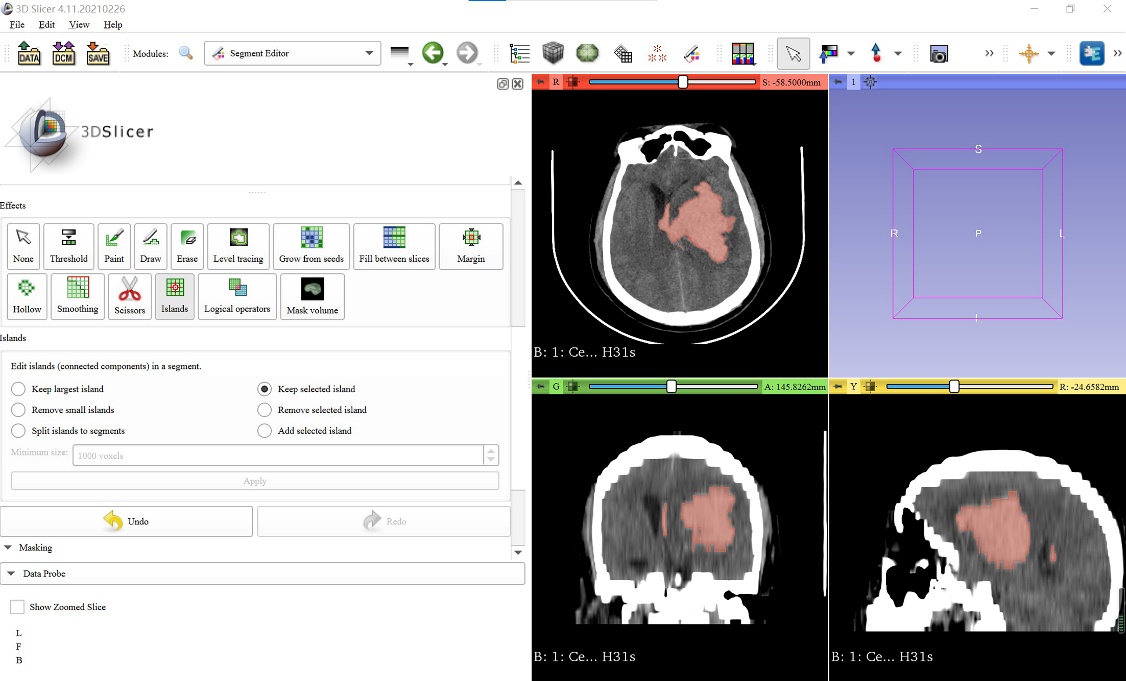


eFigure 2 Set CT threshold for hematoma segmentation


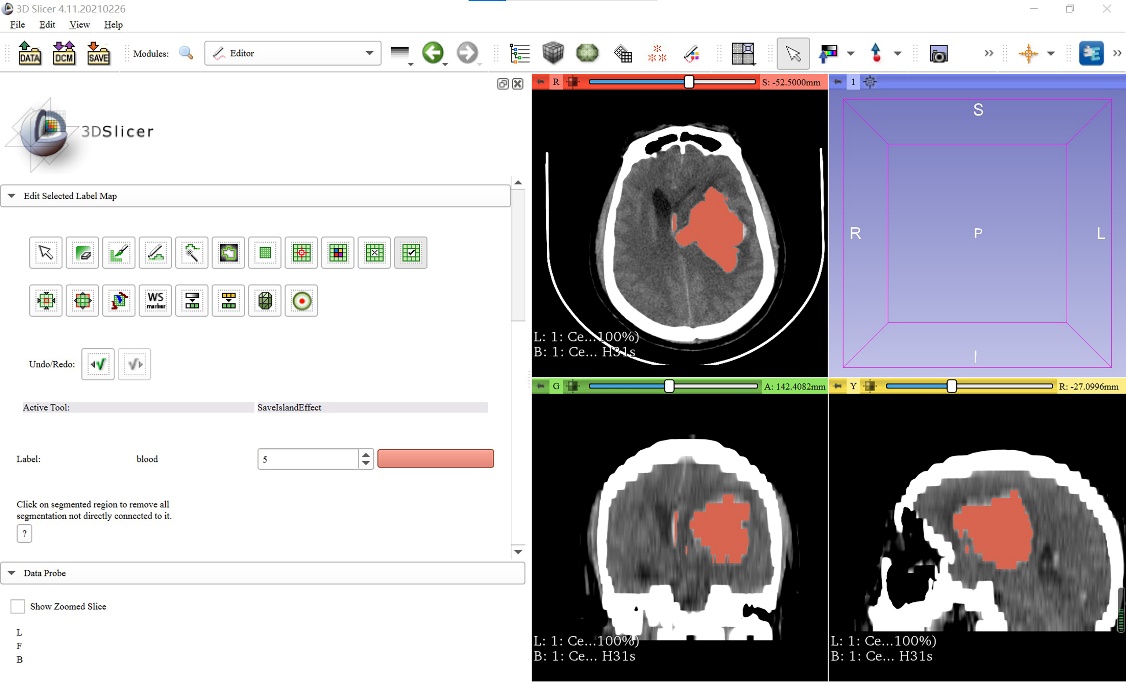


eFigure 3 Islands tool to remove non hematoma stain


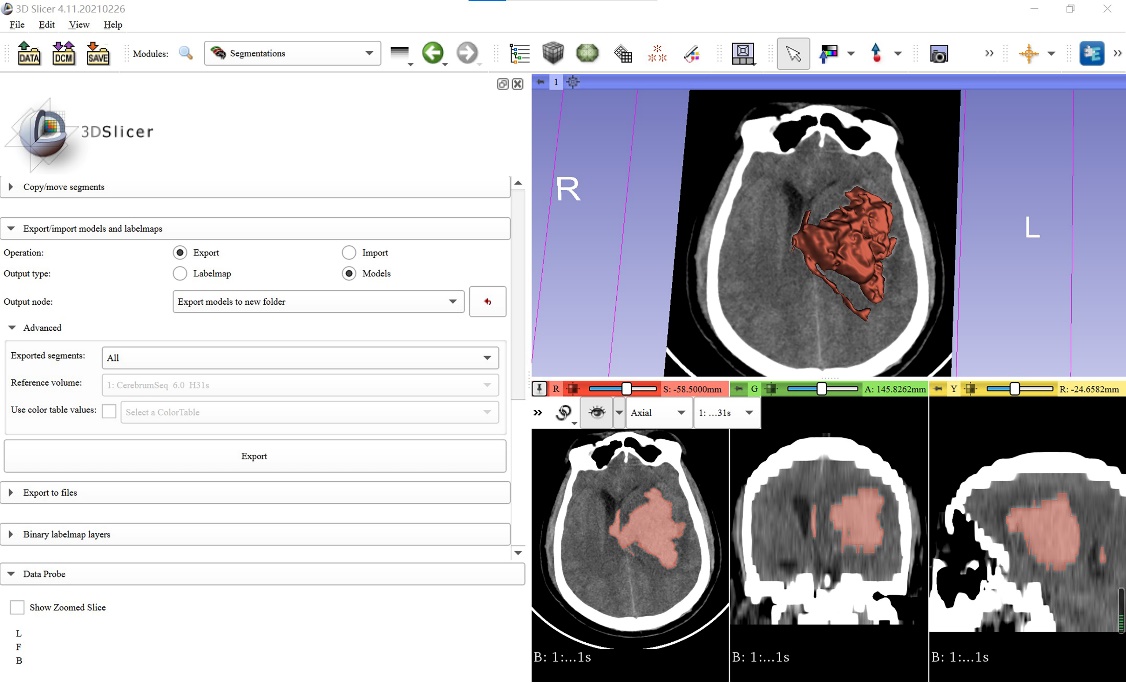


eFigure 4 Exporting 3D hematoma model in Segmentation module


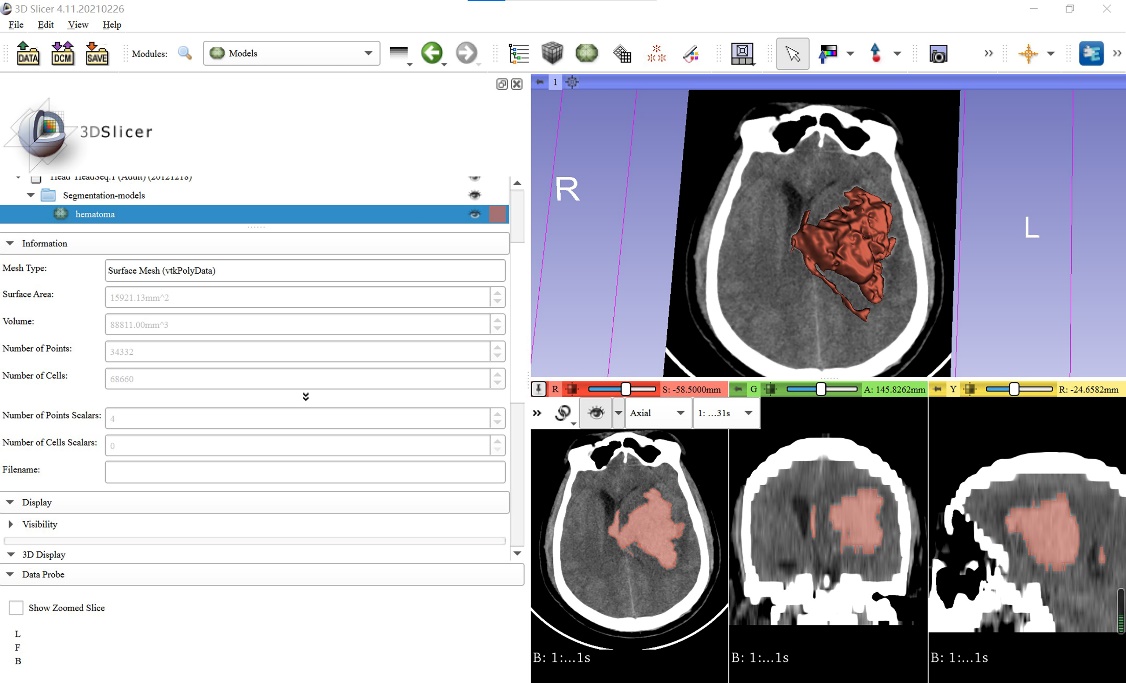


eFigure 5 Models module to view hematoma volume

There is significant estimation error using the ABC/2 formula to calculate hematoma volume, especially for irregular and multilobular hematomas. Hematoma assessment with software 3D Slicer is a low cost, accurate and helpful technique for measurement of ICH volume. In ICH clinical research, 3D Slicer can accurately calculate the volume of hematoma, which helps to improve the accuracy and reliability of research, avoid human errors, and improve the comparability and reliability of research.

**Appendix 2.** MISICH Case Report Form

**Minimally Invasive Surg****eries for Spontaneous Hypertensive Intracerebral Hemorrhage (MISICH)**

**Case Report Form**

**Research institution:**

| **01** | **Chinese PLA General Hospital** |
| --- | --- |
| **02** | **Jingzhou Central Hospital** |
| **03** | **Wuhan No.1 Hospital** |
| **04** | **Jinzhou Central Hospital** |
| **05** | **Yichang Central People’s Hospital** |
| **06** | **Hainan Hospital of Chinese PLA General Hospital** |
| **07** | **Liaoning Thrombus Hospital** |
| **08** | **Jiangmen Central Hospital** |
| **09** | **People’s Hospital of Sanshui District. Foshan** |
| **10** | **Minzu Hospital of Guangxi Medical University** |
| **11** | **The Second Hospital of Jilin University** |
| **12** | **Jilin Province People’s Hospital** |
| **13** | **Siping Central People’s Hospital** |
| **14** | **Tangdu Hospital of Air Force Military Medical University** |
| **15** | **The Third Affiliated Hospital of Inner Mongolia Medical University** |
| **16** | **The First Affiliated Hospital of Xinjiang Medical University** |

**Case Number: ______________ Enrollment Date: _____/_____/_____**

**Trial Registration Information: ClinicalTrials.gov, NCT02811614; Institution Review Board: S2016-074-01**

**Instructions:**

1. The main researchers of this trial must be trained, fully understand the trial scheme and relevant materials in advance, carry out strictly according to the plan, and select qualified cases into random groups, and should not change the grouping at will.
2. Screening qualified candidates shall fill in the formal case report form, which must be accurate and clear, and shall not be altered at will. Errors shall be corrected by drawing a double horizontal line in the centre, and the initials and time of revision shall be signed.

Example: Original record 31.8 Revision record 31.8

37.8^ZLH 2016.6.30^

3. The case numbers will be filled in by each study centre according to enrollment order and random envelope number and the pinyin abbreviation of the subject's name shall be filled in as the first letter of each word.

Example: Zhang Hong ZH ； Li Shuming LSM ；Ouyang Xiaohui OYXH

1. Filling in the □ of all selected items with “√”. For all numbers of selected grades, please mark in □.

Example: .

1. Not diagnosed (ND) should be filled in if the inspection or laboratory items are not checked or missed for some reason, and not known (NK) should be filled in if the specific situation is unknown.
2. When filling in the numbers, all □ should be filled, if there are not enough digits, fill in on the right side, and fill in the left side with “0”.

Example: 。

1. If any serious complications occur during the trial, please report to the conductor of the clinical trial in your centre in time. And the conductor of the trial centre shall report the contact person of the study project within 24 hours.

**General Patient Information**

| Case Number |  |
| --- | --- |
| Enrollment time | / / |
| Random Group Results | □ Endoscopic surgery (E)  □ Stereotactic aspiration (S)  □ Small boneflap Craniotomy (C) |
| Patient Name |  |
| Initials Abbreviation |  |
| Hospitalisation ID |  |
| Sex | □Male □Female |
| Date of Birth & Age | / /___； _____ Years old |
| Height（cm），Weight（kg） | ______cm _____kg |
| Dominant Hand | □Right □Left |
| Education Level | □Junior high school and below  □High school □Junior college  □Undergraduate □Postgraduate and above |
| Patient Relatives and Phone Number |  |
| Home Address |  |
| History and Medication of Hypertension | History：□Not □Yes， years  Medication：□Regular □Irregular □Not |
| History and Medication of Diabetes Mellitus | History：□Not □Yes， years  Medication：□Regular □Irregular □Not |
| History and Medication of Hyperlipidemia | History：□Not □Yes， years  Medication：□Regular □Irregular □Not |
| Oral Anticoagulant and Antiplatelet Drugs before Admission | □Not □Yes，name of drug：  Dosage： Time of taking： |

Responsible Physician Signature： Date： /___ / ___

**Preoperative Characteristics of Patients**

| Blood Pressure on Admission | / mmHg |
| --- | --- |
| Onset to Admission Time | hours |
| Side of Bleeding | □Left □Right |
| Location of Hematoma | □Basal Ganglia Region □Thalamus  □Ventricle □Brain Lobe， (which lobe) |
| Hematoma Volume | mL |
| State of Consciousness | □Awake □Lethargy  □Shallow Coma □Deep Coma |
| Preoperative GCS score | Eye Opening points；  Verbal Response points；  Motor Response points；  Total： points |
| Muscle strength (if applicable) | Left Upper Limb Level: ______  Left Lower Limb Level: ______  Right Upper Limb Level: _____  Right Lower Limb Level：_____ |
| Aphasia | □Yes □Not □Lack of cooperation  If yes, Type of aphasia:□Motor aphasia □Sensory aphasia □Mixed aphasia |
| CTA Examination | □Yes □Not |
| Pupil Size | Left mm； Right mm |
| Pupillary Light Reflex | Left：□Sensitive □Sluggish  Right：□Sensitive □Sluggish |

Responsible Physician Signature： Date： /___ / ___

**Intra-operative and Post-operative Information**

| Time from Onset to Operation | hours |
| --- | --- |
| Anesthetic Mode | □General Anesthesia □Local Anesthesia |
| Operation Time | hours |
| Intraoperative Blood Loss | mL |
| Intraoperative Blood Transfusion | □Not □Yes  If yes, blood transfusion volume： mL |
| Postoperative Drainage | □Not □Yes  If yes, drainage type：  □Hematoma cavity □Ventricular □Epidural |
| Postoperative Urokinase Injection | □Not □Yes  If yes， Unit； Times Per Days  Injection Time： Days |
| Hematoma volume 24-48 hours after operation | mL |
| Postoperative General Blood Pressure | / mmHg |
| GCS one week after surgery | Eye Opening points；  Verbal Response points；  Motor Response points；  Total： points |
| Re-expansion of hematoma or secondary surgery for other reasons | □Not □Yes  If yes, reason and date of secondary surgery： |
| Postoperative Intracranial Infection | □Not □Yes, Started postoperative days______, continued days_______  If yes，Cerebrospinal fluid test results: |
| Stroke-associated Pneumonia | □Not □Yes, Started postoperative days______, continued days_______  If yes，sputum bacterial culture：  □Not □Yes |

Responsible Physician Signature： Date： /___ / ___

**Evaluation at discharge**

| Total Hospitalisation Days | days |
| --- | --- |
| NICU Hospitalisation Days | days |
| Total Hospitalisation Cost | Yuan |
| GCS score at discharge | Eye Opening points；  Verbal Response points；  Motor Response points；  Total： points  **Fill in 0 in case of death!** |
| Muscle strength (if completed) | Left Upper Limb Level: ______  Left Lower Limb Level: ______  Right Upper Limb Level: _____  Right Lower Limb Level：_____ |
| Aphasia | □Yes □Not □Lack of cooperation  If yes, Type of aphasia:□Motor aphasia □Sensory aphasia □Mixed aphasia |
| Residual hematoma in head | □None □Few residues  □Large residue  Please complete at least one CT examination before discharge! |

Responsible Physician Signature： Date： /___ / ___

**One Month Follow-up**

**1、Modified Ranking Scale (mRS)：**

| Completely asymptomatic | - 0 |
| --- | --- |
| Despite several sequelae, no obvious dysfunction and can complete all daily activities. | - 1 |
| Mild disability, unable to complete all pre-illness activities, but able to complete all daily activities. | - 2 |
| Moderate disability, need some help, can walk independently | - 3 |
| Moderate and severe disability, unable to walk independently, need help in daily life | - 4 |
| Severe disability, bedridden, incontinence, total dependence on others in daily life | - 5 |
| Death | - 6 |

**2、Barthel Index (BI)：**

| Project | Scoring standard | Points |
| --- | --- | --- |
| Stool | 0=Incontinence;5=Seldom Incontinence;10=Totally controlled |  |
| Urinate | 0= Incontinence；5= Seldom Incontinence；10=Totally controlled |  |
| Shower | 0=Dependent；5=Independent |  |
| Wash Gargle | 0=Dependent；5=Independent |  |
| Dress | 0= Dependent；5= Need some help；10=Independent |  |
| To the restroom | 0= Dependent；5= Need some help；10=Independent |  |
| Eating | 0= Dependent；5= Need some help；10=Independent |  |
| Bed and chair movement | 0= Dependent，cannot sit up.；5= Need a lot of (more than 2 people) help，can sit up；10= Need a small amount of (less than 1 person) help；15= Independent |  |
| Walking | 0=cannot walk；5= independent mobility in a wheelchair；10= Need 1 person to help walk (physical or verbal guidance)；15= Walk independently (assistive device available) |  |
| Climbing stairs | 0= Dependent；5= Need some help；10=Independent |  |

Total： points；（0-100）

Responsible Physician Signature： Date： /___ / ___

**Six Months Follow-up**

**1、Modified Ranking Scale (mRS)：**

| Completely asymptomatic | - 0 |
| --- | --- |
| Despite several sequelae, no obvious dysfunction and can complete all daily activities. | - 1 |
| Mild disability, unable to complete all pre-illness activities, but able to complete all daily activities. | - 2 |
| Moderate disability, need some help, can walk independently | - 3 |
| Moderate and severe disability, unable to walk independently, need help in daily life | - 4 |
| Severe disability, bedridden, incontinence, total dependence on others in daily life | - 5 |
| Death | - 6 |

**2、Barthel Index (BI)：**

| Project | Scoring standard | Points |
| --- | --- | --- |
| Stool | 0=Incontinence;5=Seldom Incontinence;10=Totally controlled |  |
| Urinate | 0= Incontinence；5= Seldom Incontinence；10=Totally controlled |  |
| Shower | 0=Dependent；5=Independent |  |
| Wash Gargle | 0=Dependent；5=Independent |  |
| Dress | 0= Dependent；5= Need some help；10=Independent |  |
| To the restroom | 0= Dependent；5= Need some help；10=Independent |  |
| Eating | 0= Dependent；5= Need some help；10=Independent |  |
| Bed and chair movement | 0= Dependent，cannot sit up.；5= Need a lot of (more than 2 people) help，can sit up；10= Need a small amount of (less than 1 person) help；15= Independent |  |
| Walking | 0=cannot walk；5= independent mobility in a wheelchair；10= Need 1 person to help walk (physical or verbal guidance)；15= Walk independently (assistive device available) |  |
| Climbing stairs | 0= Dependent；5= Need some help；10=Independent |  |

Total： points；（0-100）

Responsible Physician Signature： Date： /___ / ___

**Case Report Form**

Declarations

As the person in charge of the trial centre, I hereby declare that: after review, the records of all items in this case report form are true, complete and accurate.

Host of trail centre signature: __________________

Date： /___ / ___

**Table S1.** Logistic regression for favorable outcome

| **Variable** | **Partial regression coefficient (B)** | **P value** | **OR** | **95% CI** |
| --- | --- | --- | --- | --- |
| Gender | -0.046 | 0.842 | 0.955 | 0.649-1.530 |
| Age | 0.045 | 0.000 | 1.046 | 1.026-1.066 |
| Preoperative GCS | -0.165 | 0.000 | 0.848 | 0.792-0.908 |
| Hematoma location | 1.818 | 0.000 | 6.159 | 3.625-10.463 |
| Hematoma volume | 0.034 | 0.000 | 1.034 | 1.018-1.051 |
| Surgery 1 (endoscopy) | -0.960 | 0.000 | 0.383 | 0.225-0.651 |
| Surgery 2 (aspiration) | -0.793 | 0.003 | 0.453 | 0.268-0.765 |
| Intracranial infection | 1.875 | 0.004 | 6.518 | 1.852-22.942 |
| Constant | -4.296 | 0.000 | 0.014 |  |
